# Electric impedance tomography-guided PEEP titration reduces mechanical power in ARDS: a randomized crossover pilot trial

**DOI:** 10.1186/s13054-023-04315-x

**Published:** 2023-01-17

**Authors:** Jose Victor Jimenez, Elizabeth Munroe, Andrew J. Weirauch, Kelly Fiorino, Christopher A. Culter, Kristine Nelson, Wassim W. Labaki, Philip J. Choi, Ivan Co, Theodore J. Standiford, Hallie C. Prescott, Robert C. Hyzy

**Affiliations:** 1 Division of Pulmonary and Critical Care Medicine, Department of Internal Medicine, University of Michigan, 1500 E Medical Center Dr. Floor 3 Reception C, Ann Arbor, MI 48109 USA; 2 UH/CVC Department of Respiratory Care, University of Michigan, Ann Arbor, MI USA; 3 VA Center for Clinical Management Research, Ann Arbor, MI USA

**Keywords:** Acute lung injury, Electrical impedance tomography, Respiratory distress syndrome, Mechanical ventilators, Ventilator-induced lung injury

## Abstract

**Background:**

In patients with acute respiratory distress syndrome undergoing mechanical ventilation, positive end-expiratory pressure (PEEP) can lead to recruitment or overdistension. Current strategies utilized for PEEP titration do not permit the distinction. Electric impedance tomography (EIT) detects and quantifies the presence of both collapse and overdistension. We investigated whether using EIT-guided PEEP titration leads to decreased mechanical power compared to high-PEEP/FiO2 tables.

**Methods:**

A single-center, randomized crossover pilot trial comparing EIT-guided PEEP selection versus PEEP selection using the High-PEEP/FiO_2_ table in patients with moderate–severe acute respiratory distress syndrome. The primary outcome was the change in mechanical power after each PEEP selection strategy. Secondary outcomes included changes in the 4 × driving pressure + respiratory rate (4 ΔP, + RR index) index, driving pressure, plateau pressure, PaO_2_/FiO_2_ ratio, and static compliance.

**Results:**

EIT was consistently associated with a decrease in mechanical power compared to PEEP/FiO_2_ tables (mean difference − 4.36 J/min, 95% CI − 6.7, − 1.95, *p* = 0.002) and led to lower values in the 4ΔP + RR index (− 11.42 J/min, 95% CI − 19.01, − 3.82, *p* = 0.007) mainly driven by a decrease in the elastic–dynamic power (− 1.61 J/min, − 2.99, − 0.22, *p* = 0.027). The elastic–static and resistive powers were unchanged. Similarly, EIT led to a statistically significant change in set PEEP (− 2 cmH_2_O, *p* = 0.046), driving pressure, (− 2.92 cmH2O, *p* = 0.003), peak pressure (− 6.25 cmH_2_O, *p* = 0.003), plateau pressure (− 4.53 cmH_2_O, *p* = 0.006), and static respiratory system compliance (+ 7.93 ml/cmH_2_O, *p* = 0.008).

**Conclusions:**

In patients with moderate–severe acute respiratory distress syndrome, EIT-guided PEEP titration reduces mechanical power mainly through a reduction in elastic–dynamic power.

*Trial registration* This trial was prospectively registered on Clinicaltrials.gov (NCT 03793842) on January 4th, 2019.

**Supplementary Information:**

The online version contains supplementary material available at 10.1186/s13054-023-04315-x.

## Introduction

In acute respiratory distress syndrome (ARDS) management, positive end-expiratory pressure (PEEP) counteracts gravity-dependent alveolar collapse, decreasing shunt and hypoxemia [1], reduces the shearing forces of cyclic alveolar opening/closing, and increases compliance [2]. Due to the heterogeneity of lung injury in ARDS, the application of PEEP can result in recruitment in some lung areas while causing overdistension in others. Suboptimal PEEP may induce ventilator-induced lung injury (VILI) [1].

Randomized controlled trials (RCTs) comparing high vs. low PEEP strategies have not consistently demonstrated the superiority of either [3]. While a network meta-analysis of 18 RCTs suggested a potential mortality benefit of higher PEEP [4], this cumulative analysis failed to consider the impact of individualized PEEP titration and the adverse effects of high PEEP on non-PEEP responders [5].

Electric impedance tomography (EIT) is a bedside imaging technique that identifies changes in lung impedance, a proxy for lung volume [6]. EIT-guided PEEP titration distinguishes PEEP-induced recruitment from overdistension [7–9]. Hse et al. demonstrated increased survival with EIT-guided PEEP titration, albeit with higher use of ECMO in the EIT group [10]. Another RCT failed to demonstrate such benefit [11].

Mechanical power (MP) is a physiological construct of the energy transmitted to the patient during invasive mechanical ventilation (IMV). MP integrates the major components of positive pressure ventilation that drive VILI [12]: elastic–static (related to PEEP), elastic–dynamic (related to driving pressure, [ΔP]), and resistive (related to flow and airway resistance). High MP is associated with ARDS mortality [13]. Given the conflicting data regarding the utility of EIT and the need for feasible surrogate endpoints to guide larger multicenter RCT, we performed a randomized crossover trial to explore the effects of EIT-guided PEEP titration on MP in patients with ARDS. We hypothesized that EIT-guided PEEP titration would result in lower MP, compared to the use of the High-PEEP/FiO_2_ table.

## Materials and methods

### Study design and population

In this single-center randomized crossover trial, we compared EIT-guided PEEP selection vs. High-PEEP/FiO_2_ tables (NCT 03793842). The University of Michigan Institutional Review Board approved this study (HUM00148126). We obtained informed consent from each patient’s legal representative. We included patients ≥ 18 years receiving IMV for ARDS management for < 72 h with a PaO_2_/FiO_2_ ratio < 150 and a PEEP > 8 cm/H_2_O. Exclusion criteria are provided in Additional file 1.

### Study protocol

Patients were randomly assigned 1:1 to receive EIT-guided PEEP titration followed by PEEP selection via the High-PEEP/FiO_2_ table (EIT first) or vice versa (tables first). Randomization was achieved using opaque, sealed envelopes. At randomization, all patients received lung-protective ventilation (LPV). PEEP was selected according to High-PEEP/FiO_2_ tables. Due to the need to maintain PEEP, a washout period was not feasible. Patients who were proned after randomization were excluded from the analysis due to its effects on PEEP requirement and MP [14].

During the EIT-guided PEEP titration phase, patients underwent a recruitment maneuver. PEEP was then decreased by two cmH_2_O every 5–10 min until a 10% drop in delta end-expiratory lung impedance in dorsal regions was detected by EIT, five cmH_2_O PEEP was reached, or hemodynamic instability/hypoxemia developed. PEEP was then set based on the intercept between the lower overdistension and collapse percentages [15].

**Fig. 1:**
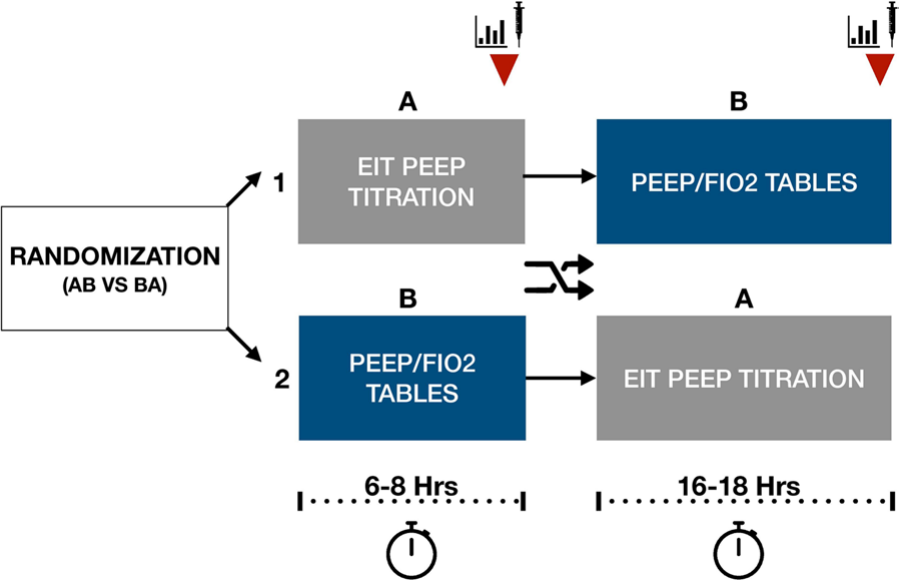
Trial design and crossover. Red arrows represent the time point at which post-intervention data were collected. Center crossed arrows represent the time of crossover

Patients randomized to the EIT-first group underwent EIT-guided PEEP titration, as above. This was followed by six hours of management per the University of Michigan ARDS protocol (Additional file 1), with PEEP left at the EIT-determined level. Afterward, patients crossed over to a PEEP level set using the High-PEEP/FiO_2_ tables, which was maintained for 14–18 h. Patients randomized to the tables-first group underwent the same interventions in the reverse order (Fig. 1). After both interventions, FiO_2_ and respiratory rate (RR) were adjusted for oxygenation > 90% and a pH 7.3–7.45, respectively.

### Outcomes

The primary outcome of this study was the change in MP after each PEEP selection strategy. Secondary outcomes included changes in the 4ΔP + RR index (an estimate of ventilator energy transfer to the lung) [13], elastic–static, elastic–dynamic, and resistive powers, as well as ΔP, plateau pressure (Pplat), PaO_2_/FiO_2_ ratio, and static compliance (Cstat). We calculated MP with Gattinoni’s simplified formula: 0.098 X RR X TV (Ppeak-[Pplat-PEEP/2]) [12] and analyzed its components.

### Statistical analysis

We compared baseline characteristics using Fisher’s test for categorical and a two-sample *t* test for continuous variables. Changes in ventilator parameters with each intervention were compared using paired t tests. We fit serial linear mixed-effect regression models assessing the association between the interventions and change in MP, adjusting for randomization order and pre-intervention MP in sequential models. We repeated this to determine the association between intervention and the 4ΔP + RR index, MP components, and ΔP. Our small sample size represents a convenience sample similar in scope to other EIT studies. Statistical analyses were performed in StataMP version 17.0 (StataCorp).

## Results

Sixteen patients were enrolled in this study. One patient was withdrawn due to hemodynamic instability; three were proned after randomization and excluded from the analysis (Additional file 2: Fig S1). Baseline characteristics are shown in Table 1. The median baseline MP was 20 J/min (IQR: 19, 28). EIT led to a significant change in PEEP compared to tables (mean difference of change: − 2 cmH_2_O, 95% CI − 3.95, − 0.05, *p* = 0.046),

**Table 1:** Baseline characteristics of participants across study arms

	All (*n* = 12)	EIT first (*n* = 6)	Tables first (*n* = 6)
Age, median [IQR], years	61 [48, 68]	62 [50, 72]	59.5 [46, 67]
Female sex, n(%)	3 (25)	1 (16)	2 (33)
White race, n(%)	11 (91)	6 (100)	5 (83)
BMI, median [IQR], kg/m^2^	32 [25, 36]	32 [26, 36]	32 [24, 36]
CCI, median [IQR]	2 (1, 5)	2 (1, 5)	2.5 (1, 5)
Tobacco Use, n(%)	9 (75)	4 (66)	5 (83)
SAPS II at ICU admission, median [IQR]	38 [33, 46]	38 [33, 45]	40 [33, 48]
SOFA at ICU admission, median [IQR]	8 (6, 9)	7 (6, 9)	8.5 (7, 10)
*Etiology of ARDS, n(%)*			
COVID-19	7 (58.3)	4 (66.7)	3 (50.0)
Bacterial pneumonia	4 (33.3)	2 (33.3)	2 (33.3)
Extrapulmonary	1 (8.3)	0 (0)	1 (16.7)
Pre-intubation NIV and/or HFNC, n(%)	11 (91.7)	6 (100)	5 (83.3)
PaO2/FiO2 Ratio, median [IQR], mmHg	130 [112, 140]	117 [111, 143]	136 [125, 138]
MV duration before inclusion, median [IQR], days	0.6 [0.2, 2]	0.5 [0.2, 2]	1 [0.2, 1]
Vasopressor at baseline, n(%)	9 (75)	5 (83.3)	4 (66.7)
Sedation at baseline (RASS), median [IQR]	− 4 [− 3, − 4.5]	− 4 [− 3, − 5]	− 3.5 [− 3, − 4]
Baseline Ventilator Settings*, median [IQR]			
Tidal Volume, ml/kg/PBW	6.0 [5.9, 6.3]	6.1 [5.7, 6.3]	6.0 [5.8, 6.3]
Respiratory rate, breaths/min	26 [20, 30]	26 [21, 30]	26 [20, 31]
PEEP, cmH_2_O	15 (14, 16)	14 (14, 16)	16 (14, 16)
Cstat, ml/cmH_2_O	36 [29, 40]	34 [28, 39]	36 [32, 42]
Ppeak, cmH_2_O	27 [25, 31]	29 [27, 35]	25 [25, 27]
Pplat, cmH_2_O	26 [25, 28]	27 [26, 28]	26 [25, 27]
Driving Pressure, cmH_2_O	11 (10, 12)	12 (12, 14)	10 (10, 11)
Mechanical Power^†^, J/min	20 [19, 28]	24 [20, 35]	19 [18, 22]
4ΔPxRR Index^§,^ J/min	70 [64, 83]	76 [68, 84]	70 [60, 73]
Non-Survivors, n(%)	6 (50)	3 (50)	3 (50)

*Baseline ventilator settings are defined as ventilator settings at the start of the study, after randomization but prior to initiation of any study intervention

^†^Mechanical Power calculate using Gattinoni’s simplified equation

^§^Mechanical Power calculated using 4ΔPxRR index

*EIT* electrical impedance tomography, *IQR* interquartile range, *SD* standard deviation, *BMI* body mass index, *CCI* Charlson Comorbidity Index, *Tobacco use* ever tobacco user (current and former smokers), *SAPS II* Simplified Acute Physiology Score II, *ICU* intensive care unit, *SOFA* Sequential Organ Failure Assessment, *ARDS* acute respiratory distress syndrome, *COVID-19* coronavirus disease 2019, *MV* mechanical ventilation, *NIV* noninvasive ventilation, *HFNC* high flow nasal cannula, *PaO2* partial pressure of oxygen, *FiO2* fraction of inspired oxygen, *RASS* Richmond Agitation Sedation Scale, *MV* mechanical ventilation, *min* minute, *PBW* predicted body weight, *PEEP* positive end-expiratory pressure, *Cstat* static respiratory system compliance, *Ppeak* peak pressure, *Pplat* plateau pressure, *J* joules

**Fig. 2:**
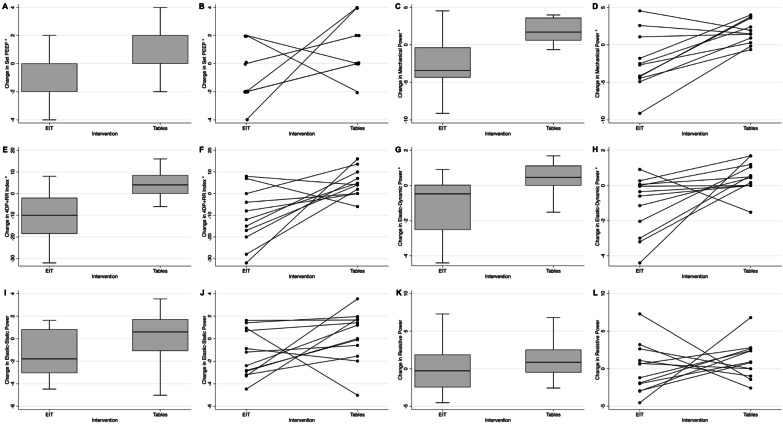
Changes in mechanical power and its components after each intervention. Changes in PEEP (**A** and **B**), MP by the Gattinoni’s Simplified formula (**C** and **D**), 4∆PxRR index (**E** and **F**), elastic–dynamic power (**G** and **H**), elastic–static power (**I** and **J**), and resistive power (**K** and **L**). Asterisk indicates a statistically significant difference in the change with the EIT versus High-PEEP tables interventions based on *p* value < 0.05

EIT decreased MP compared to PEEP/FiO_2_ tables (− 4.36 J/min, 95% CI − 6.7, − 1.95, *p* = 0.002). (Table 2). This difference persisted after adjusting for randomization order and pre-intervention MP. (Additional file 3: Tables S1-2). EIT led to a decrease in the 4ΔP + RR index (− 11.42 J/min, 95% CI − 19.01, − 3.82, *p* = 0.007) mainly through a decrease in elastic–dynamic power (− 1.61 J/min, 95% CI: − 2.99, − 0.22, *p* = 0.027), and driving pressure (− 2.92 J/min, 95% CI: − 4.59, − 1.23, *p* = 0.003) (Table 2 and Fig. 2). These differences persisted after adjusting for randomization order and baseline MP (Additional file 3: Table S3). Elastic–static and resistive powers were unchanged across both interventions.

**Table 2:** Comparison of changes in ventilator parameters with EIT vs tables, for all participants, *n* = 12

	Change with EIT*	Change with tables**		95% CI of mean difference	*p* value
Mechanical Power^1^, J/min	− 2.50 ± 3.70	1.87 ± 1.61	− 4.36	(− 6.77, − 1.95)	0.002
4ΔP + RR Index, J/min	− 6.80 ± 9.36	4.62 ± 6.25	− 11.42	(− 19.01, − 3.82)	0.007
Elastic–static power^2^, J/min	− 1.37 ± 2.11	0.19 ± 2.28	− 1.56	(− 3.71, 0.58)	0.138
Elastic–dynamic power^3^, J/min	− 1.13 ± 1.66	0.48 ± 0.88	− 1.61	(− 2.99, − 0.22)	0.027
Resistive power^4^, J/min	0.01 ± 3.30	1.15 ± 2.48	− 1.14	(− 4.59, 2.30)	0.48
Driving Pressure, cmH_2_O	− 1.58 ± 2.32	1.34 ± 1.31	− 2.92	(− 4.59, − 1.24)	0.003
PEEP (set), cmH_2_O	− 1.17 ± 1.80	0.83 ± 1.80	− 2	(− 3.95, − 0.05)	0.046
Ppeak, cmH_2_O	− 2.75 ± 3.55	3.5 ± 2.78	− 6.25	(− 9.79, − 2.71)	0.003
Pplat, cmH_2_O	− 2.48 ± 3.22	2.06 ± 1.88	− 4.53	(− 7.45, − 1.62)	0.006
RR, breaths/min	− 0.5 ± 2.35	− 0.75 ± 2.73	0.25	(− 2.71, 3.21)	0.856
Cstat, ml/cmH_2_O	3.24 ± 9.85	− 4.6 ± 5.26	7.93	(2.54, 13.32)	0.008
PaO2/FiO2 ratio	25.14 ± 27.11	− 0.89 ± 60.05	26.03	(− 16.01, 68.06)	0.2

Data are listed as mean ± SD

*P* value calculated using paired *t* test

*Change with EIT: ventilator parameter at the end of EIT intervention minus ventilator parameter at the start of the EIT intervention

**Change with tables: ventilator parameter at the end of the table intervention minus ventilator parameter at the start of the table intervention

^1^Mechanical Power calculate using Gattinoni’s simplified equation

^2^Elastic–static power, related to PEEP (J/min) = 0.098 * VT * RR * PEEP

^3^Elastic–dynamic power, related to DP (J/min) = 0.0983 * VT * RR * 0.5 * DP

^4^Resistive power, related to resistance in the ventilator circuit, endotracheal tube, and airways (J/min) = 0.098 * VT * RR *(Ppeak—Pplat)

*EIT* Electrical Impedance Topography, *J* joules, *min* minute, *PEEP* positive end-expiratory pressure, *Ppeak* peak pressure, *Pplat* plateau pressure, *RR* respiratory rate, *Cstat* static respiratory system compliance, *PaO2* partial pressure of oxygen, *FiO2* fraction of inspired oxygen, *SD* = standard deviation

EIT led to changes in peak pressures (− 6.25 cmH_2_O, *p* = 0.003), Pplat (− 4.53 cmH_2_O, *p* = 0.006), and Cstat (+ 7.93 ml/cmH_2_O, *p* = 0.008) (Additional file 4: Fig S2). There was no significant change in RR or PaO_2_/FiO_2_ ratio.

After the EIT phase, one patient developed pneumomediastinum, which did not require additional intervention. Three patients developed hypotension during the RM. In one patient, the protocol was stopped due to persistent hemodynamic instability.

## Discussion

In this randomized crossover trial, we found a significant decrease in MP using EIT-guided PEEP titration compared to High-PEEP/FiO_2_ tables in mechanically ventilated patients with moderate–severe ARDS. This difference persisted after sensitivity analysis and adjustment for randomization order and pre-intervention MP. A reduction in the elastic–dynamic MP mainly drove the decrease in MP.

Zhao and colleagues reported that EIT-guided PEEP titration was associated with improved respiratory mechanics [15]. Similarly, a RCT by Hsu and colleagues reported improvement in ΔP, Cstat, and survival rates with EIT-guided PEEP titration compared to the pressure–volume curves through a decrease in PEEP [11].

He and colleagues compared the effects of EIT-guided PEEP titration vs. a low PEEP/FiO_2_ table [10] without finding differences in survival, ventilator-free days, or ICU stay. However, this study was limited by using similar PEEP between groups and including mild ARDS. In our study, EIT-guided PEEP titration led to significant changes in PEEP, and we only enrolled patients with moderate–severe ARDS. Using a crossover design allowed us to analyze the effects of each intervention on an individual level by using each patient as their own control. Using the High-PEEP/FiO_2_ tables as the control intervention permitted comparison with the strategy associated with better ventilation/perfusion matching [16] and outcomes in severe ARDS [4].

In patients with ARDS, persistent elevation of MP > 17 J/min is associated with higher mortality [13, 17]. Although patients in our study received standard LPV per protocol at baseline, MP was elevated (median 20 J/min). EIT-guided PEEP titration led to a mean reduction of MP by 4.36 J/min, a reduction associated with decreased mortality [17, 18], particularly when achieved during the initial hours of IMV [18]. The reduction in MP was achieved through decreased elastic–dynamic power, but not the elastic–static or resistive powers. Although changes in RR were allowed to achieve pH > 7.3, they were not different across groups.

Our findings are consistent with previous observations [13] that propose oscillating mechanical stresses as the main injurious mechanism for VILI. In our study, EIT decreased PEEP levels despite ∆Ps believed to be LPV, suggesting EIT-directed PEEP titration to be a more effective means of optimizing LPV than PEEP/FiO2 tables via a reduction in MP. In addition, observational studies have shown that lung recruitment is not systematically associated with detectable improvements in Cstat nor ∆P, therefore precluding accurate titration of PEEP based exclusively on these parameters [19, 20].

Our study has several limitations; 1) excluding subjects on prone positioning could have introduced post-randomization selection bias. 2) Sample was small, but the effects in MP reduction were significant and occurred despite optimal LPV at baseline. This suggests a strong effect of EIT in reducing MP. 3) We did not include a washout phase. However, our analysis considered the order of interventions to assess for carryover effects. 4) We did not assess for recruitability before the recruitment maneuver. This could have impacted sample’s enrichment. 5) Our intervention focused on titrating PEEP during the initial 24 h. However, PEEP/FiO_2_ tables are meant to guide continuous changes in PEEP based on FiO_2_ responses, dead space fraction, and mechanics. This was not assessed. 6) MP was calculated using airway not transpulmonary ΔP rather which could have introduced measurement bias.

## Conclusion

This study shows that EIT-guided PEEP titration decreases MP in patients with moderate–severe ARDS compared to a high-PEEP/FiO2 table. A decrease in the dynamic–elastic component primarily drives the reduction in MP. The clinical impact of EIT-guided PEEP titration should be tested in large multicenter trials.

## Supplementary Information

**Additional file 1. **Extended methods and protocol.

**Additional file 2. **Flow diagram of the study.

**Additional file 3.** Comparison of ventilator parameters.

**Additional file 4.** Changes in respiratory mechanics.

## Data Availability

The datasets used and analyzed in this study are available from the corresponding author upon reasonable request.

## Abbreviations

ARDS: Acute respiratory distress syndrome
Cstat: Static compliance
EIT: Electric impedance tomography
FiO2: Fraction of inspired oxygen
IMV: Invasive mechanical ventilation
MP: Mechanical power
PEEP: Positive end-expiratory pressure
Ppeak: Peak pressure
Pplat: Plateau pressure
RCTs: Randomized controlled trials
RR: Respiratory rate
TV: Tidal volume
VILI: Ventilator-induced lung injury
∆P: Driving pressure
