# Supplementary material for: Electric impedance tomography-guided PEEP titration reduces mechanical power in ARDS: a randomized crossover pilot trial
Source: Crit Care. 2023 Jan 17;27:21. doi: 10.1186/s13054-023-04315-x (PMC9843117; doi:10.1186/s13054-023-04315-x)
Supplement: Supplementary file 1 — Additional file 1. Extended methods and protocol. [file 13054_2023_4315_MOESM1_ESM.docx]

***Study Protocol Supplement***

***Full list of Inclusion/Exclusion Criteria***

## **Inclusion Criteria**

1. Age > 18 years
2. Endotracheal ventilation for < 1 week (168 hours)
3. Presence of all of the following conditions for < 72 hours
   - 1. PaO2/FiO2 < 150 with PEEP > 5 cm H2O for > 30 min.

OR, IF ABG NOT AVAILABLE

SpO2/FiO2 ratio that is equivalent to a PaO2/FiO2 < 150 with PEEP > 8 cm H2O (Appendix G), and a confirmatory SpO2/FiO2 ratio between 1-6 hours after the initial SpO2/FiO2 ratio determination

- - 1. respiratory failure not fully explained by cardiac failure or fluid overload

1. Bilateral opacities not fully explained by effusions, lobar/lung collapse, or nodules for < 1 week (168 hours)
2. All criteria listed in (3) developed within 1 week of a known clinical insult or new or worsening respiratory symptoms

The 72-hour enrollment time window begins when criteria 1-4 are met. Criteria may be met at either the University of Michigan Hospital or a referring hospital.

## **Exclusion Criteria**

1. Lack of informed consent
2. Known pregnancy
3. ECMO
4. Severe chronic respiratory disease requiring home oxygen therapy or ventilation
5. Calculated BMI of greater than 50
6. Severe chronic liver disease defined as a Child-Pugh score of 12-15
7. Prior bone marrow transplantation or present chemotherapy induced neutropenia
8. Expected duration of mechanical ventilation of < 48 hours
9. Decision to withhold life-sustaining treatment
10. Moribund patient not expected to survive 24 hours
11. Diffuse alveolar hemorrhage from vasculitis
12. Burns > 40% total body surface or burns on the thorax
13. Unwillingness to utilize the ARDS Network 6 ml/kg IBW ventilation protocol
14. Neurologic conditions with risk of intracranial hypertension
15. ARDS criteria met for > 48 hours
16. Contraindications to using EIT (Presence of a pacemaker or AICD, inability to place the belt (presence of surgical wounds dressing, thoracic or spinal cord trauma, recent thoracic surgery, etc), undrained pneumothorax or BPF)
17. Platelet count < 50 K/uL

### **Exclusion Definitions**

#### **1. Child-Pugh Score (Pugh, 1973)**

Points Class

5-6 A

7-9 B

≥ 10 C

|  | Numerical Score for Increasing Abnormality | | |
| --- | --- | --- | --- |
| Measurement | 1 | 2 | 3 |
| Ascites | None | Present | Tense |
| Encephalopathy | None | Grade I or II | Grade III or IV |
| Bilirubin (mg/dl) | < 2 | 2-3 | > 3 |
| Albumin (g/L) | > 35 | 28-35 | < 28 |
| Prothrombin time (sec. prolonged) | 1-4 | 4-10 | > 10 |

#### **2. Severe Chronic Respiratory Disease**

Any of the following is considered severe chronic respiratory disease and excludes a patient from being eligible for enrollment:

1. FEV_1_ less than 20 ml/kg PBW (e.g. 1.4 L for a 70 kg person), or
2. FEV_1_/VC less than 50% predicted, or
3. Chronic hypercapnia (PaCO_2_ greater than 45 mmHg) and/or chronic hypoxemia (PaO_2_ less than 55 mmHg) on F_I_O_2_ = 0.21, or
4. Radiographic x-ray evidence of any chronic over-inflation or chronic interstitial infiltration, or
5. Hospitalization within the past six months for respiratory failure in patients with chronic respiratory disease. (PaCO_2_ greater than 50 mmHg or PaO_2_ less than 55 mmHg or O_2_-Sat < 88% on FiO_2_ = .21).
6. Chronic restrictive, obstructive, neuromuscular, chest wall or pulmonary vascular disease resulting in severe exercise restriction, e.g., unable to climb stairs or perform household duties, secondary polycythemia, severe pulmonary hypertension (mean PAP greater than 40 mmHg), or ventilator dependency.

***Protocol (Expanded)***

***EIT-guided PEEP titration***

During the EIT-guided PEEP titration, a 16-electrode silicone EIT belt was positioned along the fourth to fifth intercostal space and then connected to the EIT monitor (Pulmovista 500; Dräger, Lübeck, Germany). After electrode stabilization and calibration, PEEP titration and EIT measurements were performed.

Recruitment Phase: After ensuring hemodynamic stability (mean arterial pressure ≥ 65 mmHg monitored by arterial line with or without vasopressor support), patients were placed on pressure-control continuous mandatory ventilation (PC-CMV). FiO2, TV and respiratory rate (RR) were left unchanged. PEEP was progressively titrated in increments of 3 cmH_2_O every two minutes until reaching 20 cmH_2_O, after which a delta pressure of 15 cm H2O was applied (35/20) for 5-10 breaths. If hemodynamic stability was maintained, PEEP was then increased to 25 cm H_2_O (i.e., 40/25) for 1 minute. If patients developed/worsened hypotension (mean arterial pressure < 65 mmHg or 20% decrease) or hypoxemia (< 88% SaO_2_ with unchanged FiO_2_), the recruitment phase was terminated, and PEEP was set to the previous level in which stability was maintained.

Decremental Phase: After the recruitment phase, patients continued in PC-CMV, and PEEP was decreased by 2 cm H_2_O every 5-10 minutes until one of the following occurred: 1) a 10% drop in delta end-expiratory lung impedance (ΔEELI), expressed as tidal variation, in any region of interest (ROI) after a stabilization period in the same PEEP level (15) 2) PEEP of 5 cmH_2_O was reached, or 3) patient developed hemodynamic instability or SpO_2_ < 88%.

PEEP was selected based on the intercept between the lower overdistension and collapse (ODCL) percentages (15). If the intercept point occurred between two PEEP values, we selected the one with the lowest sum of ODCL. If the ODCL sum was equal, then the PEEP with the lowest regional ventilation delay was chosen (30).

***Protocols By Study Group***

EIT-first group: After the measurement of baseline parameters, patients randomized to the EIT-first group underwent EIT-guided PEEP titration, as above. For the next six hours, ventilator management followed the University of Michigan ARDS protocol **(Supplementary Digital Content)**, except PEEP was left at the EIT-determined level, and only FiO_2_ was adjusted for oxygenation issues. After six hours, patients crossed over to a PEEP level set using the high PEEP/FiO_2_ tables protocol, This PEEP was maintained for an additional 14-18 hours, and only FiO_2_ was adjusted for oxygenation issues.

PEEP tables-first group: After measurement of baseline parameters, patients randomized to the High-PEEP/FiO_2_ tables-first group were set on a PEEP level determined by the high PEEP/FiO_2_ table protocol for the initial six hours of the study. During these six hours, ventilator management followed the University of Michigan ARDS protocol**,** except PEEP, which was left at the table-determined level, and only FiO_2_ was adjusted for oxygenation issues. Afterward, patients were crossed-over to the EIT-guided PEEP titration, using the protocol described above. The selected PEEP was maintained for the next 16-18 hours, and only FiO_2_ was adjusted for oxygenation issues.

### **ARDS Ventilator Management Strategies**


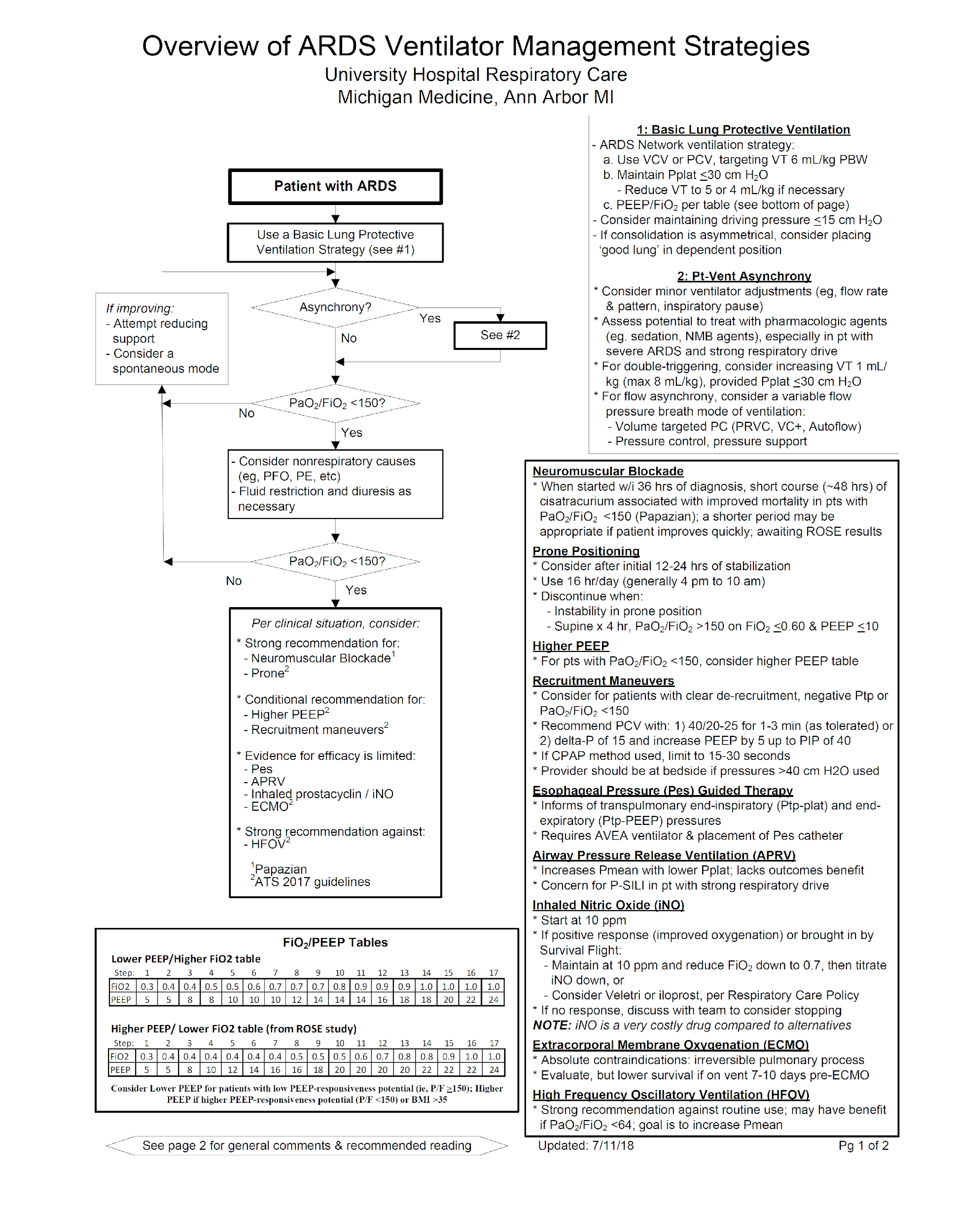


**Figure S1: Flow Diagram**

**Figure S2: Changes in respiratory mechanics**

Changes in driving pressure **(A and B)**, plateau pressure **(C and D)**, peak pressure **(E and F)**, respiratory rate **(G and H),** static compliance **(I and J)** and PaO2/FiO2 ratio **(K and L)**

|  |  |  |
| --- | --- | --- |
|  | | |
|  | | |
|  | | |
|  |  |  |
|  |  |  |
