# Supplementary figures and images for: Electric impedance tomography-guided PEEP titration reduces mechanical power in ARDS: a randomized crossover pilot trial
Source: Crit Care. 2023 Jan 17;27:21. doi: 10.1186/s13054-023-04315-x (PMC9843117; doi:10.1186/s13054-023-04315-x)

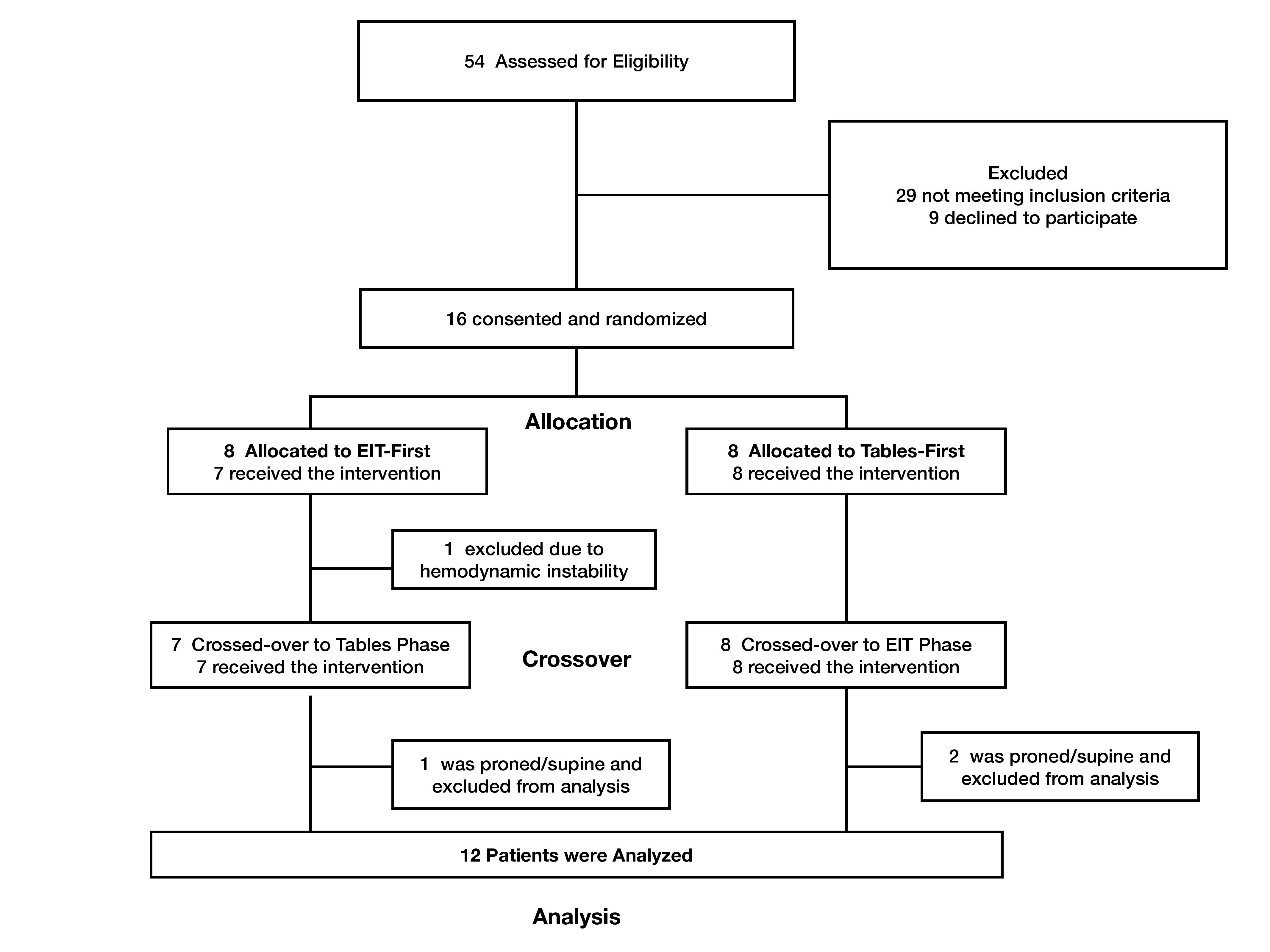

Supplement: Supplementary file 2 — Additional file 2. Flow diagram of the study. [file 13054_2023_4315_MOESM2_ESM.jpeg]

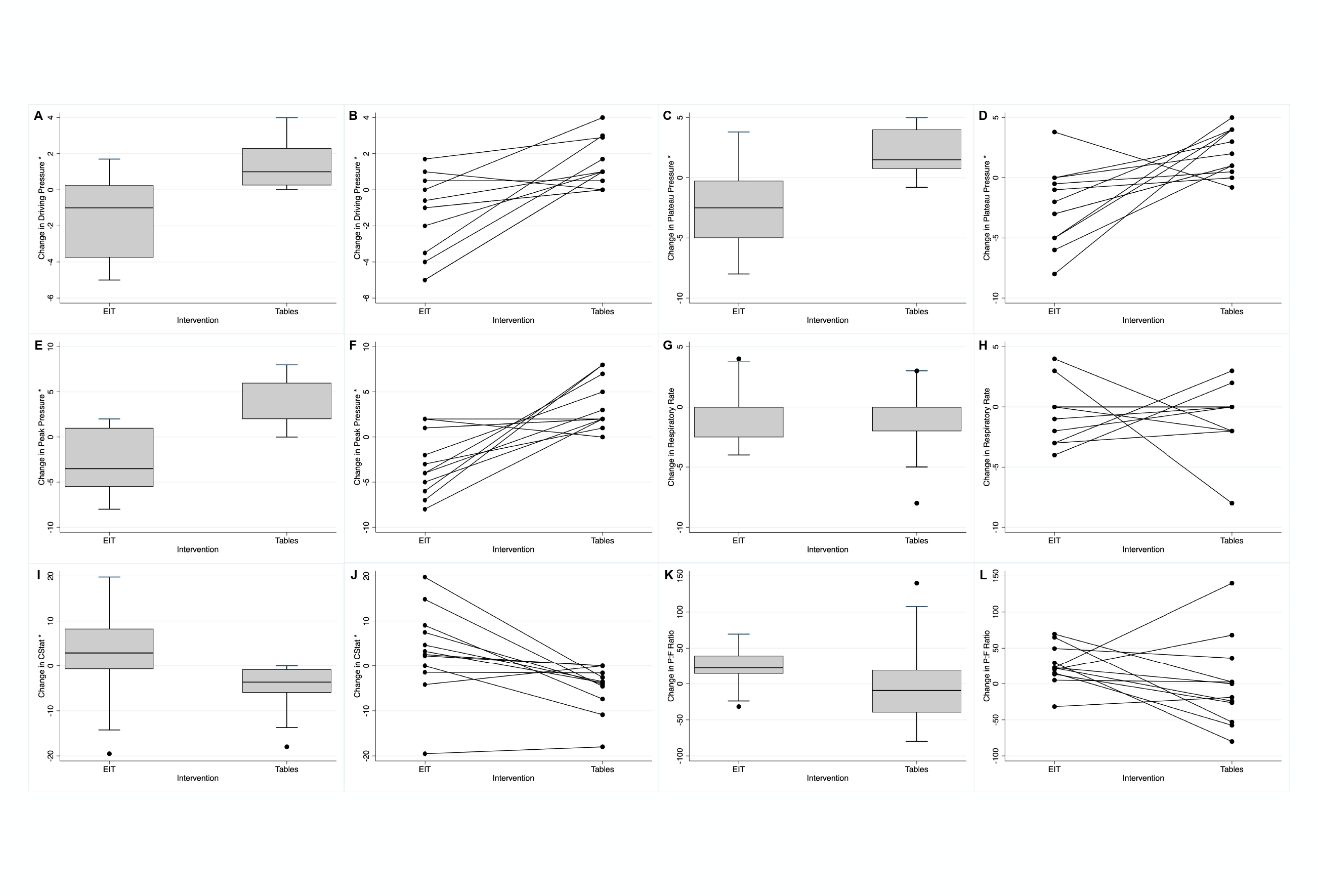

Supplement: Supplementary file 4 — Additional file 4. Changes in respiratory mechanics. [file 13054_2023_4315_MOESM4_ESM.jpeg]
