# Supplementary material for: Electric impedance tomography-guided PEEP titration reduces mechanical power in ARDS: a randomized crossover pilot trial
Source: Crit Care. 2023 Jan 17;27:21. doi: 10.1186/s13054-023-04315-x (PMC9843117; doi:10.1186/s13054-023-04315-x)
Supplement: Supplementary file 3 — Additional file 3. Comparison of ventilator parameters. [file 13054_2023_4315_MOESM3_ESM.docx]

|  |  |  |  |  |  |
| --- | --- | --- | --- | --- | --- |
|  |  |  |  |  |  |
| **Supplementary Table 1:** Comparison of ventilator parameters after EIT vs Tables, n=12. | | | | | |
|  | | | | | |
|  | | | **Post-EIT** | **Post-Tables** | **p-value** |
| Mechanical Power^1^, J/min | | | 21.77 ± 6.53 | 23.52 ± 4.50 | 0.056 |
| 4ΔP+RR, J/min | | | 67.18 ± 9.15 | 72.63 ± 9.98 | 0.043 |
| Elastic-static power^2^, J/min | | | 14.03 ± 3.62 | 14.75 ± 3.31 | 0.24 |
| Elastic-dynamic power^3^, J/min | | | 5.09 ± 1.38 | 5.86 ± 2.0 | 0.08 |
| Resistive power^4^, J/min | | | 2.64 ± 2.83 | 2.91 ± 1.88 | 0.77 |
| Driving Pressure, cmH_2_O | | | 10.6 ± 1.82 | 11.91 ± 1.57 | 0.026 |
| PEEP (set), cmH_2_O | | | 14 ± 2.83 | 15.17 ± 3.24 | 0.067 |
| PPeak, cmH_2_O | | | 27.67 ± 3.11 | 30.42 ± 2.68 | 0.004 |
| Pplat, cmH_2_O | | | 25.32 ± 2.84 | 27.38 ± 2.87 | 0.004 |
| RR, breaths/min | | | 24.75 ± 6.43 | 25 ± 6.40 | 0.667 |
| CStat, ml/cmH_2_O | | | 39.56 ± 10.91 | 34.62 ± 7.68 | 0.039 |
| PaO2/FiO2 ratio | | | 163.10 ± 43.69 | 152.70 ± 66.18 | 0.497 |
| **Table Legend**  Data are listed as mean ± SD.  P-value calculated using paired t-test.  1 Mechanical Power calculate using Gattinoni´s simplified equation  2 Elastic–static power, related to PEEP (J/min) = 0.098 * VT * RR * PEEP. 3 Elastic–dynamic power, related to DP (J/min) = 0.0983 * VT * RR * 0.5 * DP. 4 Resistive power, related to resistance in the ventilator circuit, endotracheal tube, and airways(J/min) = 0.098 * VT * RR *(Ppeak - Pplat).  **Definitions of Abbreviations:** EIT= Electrical Impedance Topography, J=joules, min=minute, PEEP= positive end-expiratory pressure, Ppeak= peak pressure, Pplat= plateau pressure, RR= respiratory rate, Cstat= static respiratory system compliance, PaO2= partial pressure of oxygen, FiO2= fraction of inspired oxygen, SD= standard deviation | | | | | |
|  |  |  |  |  |  |
|  |  |  |  |  |  |

**Supplementary 2.** Serial linear mixed effect models assessing the association between intervention (EIT vs table) and change in mechanical power (MP).

| **Table 2a.** Association between intervention (EIT vs Table) and change in mechanical power (MP) calculated by Gattinoni´s Simplified Equation | | | | | |
| --- | --- | --- | --- | --- | --- |
|  | **Coefficient** | **95% CI** | **P value** | **Mean Predicted Change in MP, Joules/min** | **95% CI** |
| **Model 1:** no covariates | | | | | |
| Intervention |  |  |  |  |  |
| Tables | ref |  |  | 1.87 | (0.32, 3.41) |
| EIT | -4.36 | (-6.42, -2.31) | <0.001 | -2.50 | (-4.04, -0.95) |
| **Model 2**: randomization order as a covariate | | | | | |
| Intervention |  |  |  |  |  |
| Tables | ref |  |  | 1.87 | (0.34, 3.39) |
| EIT | -4.36 | (-6.42, -2.31) | <0.001 | -2.50 | (-4.02, -0.97) |
| Randomization order |  |  |  |  |  |
| Tables First | ref |  |  | 0.12 | (-1.48, 1.71) |
| EIT First | -0.86 | (-3.11, 1.39) | 0.454 | -0.75 | (-2.33, 0.85) |
| **Model 3**: randomization order and pre-intervention MP as covariates | | | | | |
| Intervention |  |  |  |  |  |
| Tables | ref |  |  | 1.62 | (0.16, 3.09) |
| EIT | -3.88 | (-5.83, -1.93) | < 0.001 | -2.25 | (-3.72, -0.79) |
| Randomization order |  |  |  |  |  |
| Tables First | ref |  |  | -0.28 | (-1.89, 1.32) |
| EIT First | -0.06 | (-2.41, 2.29) | 0.958 | -0.35 | (-1.95, 1.26) |
| Pre-intervention MP* | -0.19^§^ | (-0.39, 0.02) | 0.074 | n/a | n/a |
| 15 J/min |  |  |  | 1.16 | (-0.79, 3.11) |
| 20 J/min |  |  |  | 0.23 | (-1.01, 1.48) |
| 25 J/min |  |  |  | -0.69 | (-1.86, 0.47) |
| 30 J/min |  |  |  | -1.62 | (-3.42, 0.18) |
| 35 J/min |  |  |  | -2.55 | (-5.22, 0.13) |
| **Table 2a Legend**  *Pre-intervention MP (mechanical power) was determined by calculating the MP before each intervention, using Gattinoni´s Simplified formula  ^§^ Coefficient of change in MP during intervention per 1 J/min difference in pre-intervention MP  EIT= Electrical Impedance Topography, CI = confidence interval, MP= mechanical power, J=Joules, min=minute | | | | | |

| **Table 2b.** Association between intervention (EIT vs Table) and change in 4ΔPxRR Index | | | | | |
| --- | --- | --- | --- | --- | --- |
|  | **Coefficient** | **95% CI** | **P value** | **Mean Predicted Change in MP, J/min** | **95% CI** |
| **Model 1:** no covariates |  |  |  |  |  |
| Intervention |  |  |  |  |  |
| Tables | ref |  |  | 4.62 | (0.31, 8.93) |
| EIT | -11.42 | (-17.51, -5.32) | <0.001 | -6.80 | (-11.12, -2.49) |
| **Model 2**: randomization order as a covariate | | | | | |
| Intervention |  |  |  |  |  |
| Tables | ref |  |  | 4.62 | (0.31, 8.92) |
| EIT | -11.42 | (-17.51, -5.33) | <0.001 | -6.80 | (-11.11, -2.49) |
| Randomization order |  |  |  |  |  |
| Tables First | ref |  |  | -0.83 | (-5.14, 3.47) |
| EIT First | -0.52 | (-6.61, 5.57) | 0.868 | -1.35 | (-5.66, 2.96) |
| **Model 3**: randomization order and pre-intervention MP as covariates | | | | | |
| Intervention |  |  |  |  |  |
| Tables | ref |  |  | 3.40 | (0.04, 6.76) |
| EIT | -8.97 | (-13.80, -4.15) | <0.001 | -5.58 | (-8.94, -2.22) |
| Randomization order |  |  |  |  |  |
| Tables First | Ref |  |  | -0.66 | (-3.97, 2.65) |
| EIT First | -0.86 | (-5.54, 3.82) | 0.719 | -1.52 | (-4.83, 1.79) |
| Pre-intervention 4ΔPxRR | -0.41^§^ | (-0.61, -0.21) | <0.001 |  |  |
| 50 J/min |  |  |  | 7.51 | (2.76, 12.25) |
| 60 J/min |  |  |  | 3.41 | (0.23, 6.60) |
| 70 J/min |  |  |  | -0.68 | (-3.03, 1.67) |
| 80 J/min |  |  |  | -4.78 | (-7.71, -1.84) |
| 90 J/min |  |  |  | -8.87 | (-13.28, -4.46) |
|  |  |  |  |  |  |
|  | | | | | |

| **Table 2c.** Association between intervention (EIT vs Table) and change in driving pressure (DP) | | | | | |
| --- | --- | --- | --- | --- | --- |
|  | **Coefficient** | **95% CI** | **P value** | **Mean Predicted Change in DP, cmH_2_O** | **95% CI** |
| **Model 1:** no covariates |  |  |  |  |  |
| Intervention |  |  |  |  |  |
| Tables | ref |  |  | 1.34 | (0.32, 2.36) |
| EIT | -2.92 | (-4.35, -1.49) | <0.001 | -1.58 | (-2.59, -0.56) |
| **Model 2**: randomization order as a covariate | | | | | |
| Intervention |  |  |  |  |  |
| Tables | ref |  |  | 1.34 | (0.33, 2.36) |
| EIT | -2.92 | (-4.35, -1.49) | <0.001 | -1.58 | (-2.59, -0.56) |
| Randomization order |  |  |  |  |  |
| Tables First | ref |  |  | 0.04 | (-0.98, 1.06) |
| EIT First | -0.32 | (-1.76, 1,13) | 0.667 | -0.28 | (-1.30, 0.75) |
| **Model 3**: randomization order and pre-intervention driving pressure (DP) | | | | | |
| Intervention |  |  |  |  |  |
| Tables | ref |  |  | 0.86 | (0.10, 1.63) |
| EIT | -1.96 | (-3.07, -0.84) | 0.001 | -1.09 | (-1.86, -0.33) |
| Randomization order |  |  |  |  |  |
| Tables First | ref |  |  | 0.24 | (-0.51, 0.99) |
| EIT First | -0.71 | (-1.77, 0.35) | 0.188 | -0.47 | (-1.22, 0.27) |
| Pre-intervention DP | -0.59^§^ | (-0.85, -0.34) | <0.001 |  |  |
| 5 cmH_2_O |  |  |  | 3.67 | (1.99, 5.36) |
| 10 cmH_2_O |  |  |  | 0.70 | (0.07, 1.33) |
| 15 cmH_2_O |  |  |  | -2.27 | (-3.32, -1.22) |
| **Table 2c Legend**  ^§^ Coefficient of change in DP during intervention per 1 cmH_2_O difference in pre-intervention DP  EIT= Electrical Impedance Topography, CI = confidence interval, DP= driving pressure | | | | | |

**Table 3. Association between intervention (EIT vs Table) and change in elastic-dynamic power**

|  | **Coefficient** | **95% CI** | **P value** | **Mean Predicted Change in MP, Joules/min** | **95% CI** |
| --- | --- | --- | --- | --- | --- |
| **Model 1:** no covariates | | | | | |
| Intervention |  |  |  |  |  |
| Tables | ref |  |  | 0.48 | (-0.24, 1.20) |
| EIT | -1.61 | (-2.62, -0.59) | 0.002 | -1.13 | (-1.85, -0.41) |
| **Model 2**: randomization order as a covariate | | | | | |
| Intervention |  |  |  |  |  |
| Tables | ref |  |  | 0.48 | (-0.24, 1.20) |
| EIT | -1.61 | (-2.62, -0.59) | 0.002 | -1.13 | (-1.85, -0.41) |
| Randomization order |  |  |  |  |  |
| Tables First | ref |  |  | -0.29 | (-1.01, 0.42) |
| EIT First | -0.06 | (-1.08, 0.95) | 0.9 | -0.36 | (-1.07, 0.36) |
| **Model 3**: randomization order and pre-intervention MP as covariates | | | | | |
| Intervention |  |  |  |  |  |
| Tables | ref |  |  | 0.32 | (-0.23, 0.87) |
| EIT | -1.28 | (-2.07, -0.5) | 0.001 | -0.97 | (-1.52, -0.42) |
| Randomization order |  |  |  |  |  |
| Tables First | ref |  |  | -0.39 | (-0.93, 0.16) |
| EIT First | 0.13 | (-0.64, 0.90) | 0.74 | -0.26 | (-0.81, 0.28) |
| Pre-intervention dynamic pwr | -0.39^§^ | (-0.56, -0.21) | <0.001 | n/a | n/a |
| 2.5 J/min |  |  |  | 0.95 | (0.24, 1.65) |
| 5 J/min |  |  |  | -0.02 | (-0.43, 0.39) |
| 7.5 J/min |  |  |  | -0.98 | (-1.47. -0.49) |
| 10 J/min |  |  |  | -1.94 | (-2.79, -1.10) |

**Supplementary 3: Parameters of PEEP down titration**

| subject | Starting PEEP (EIT titration) | Lowest Peep (EIT titration) | Final PEEP (EIT titration) | Reason titration was stopped |
| --- | --- | --- | --- | --- |
| 1 | 20 | 10 | 16 | >10% drop in delta EELI ROI 4 (dorsal) |
| 2 | 20 | 14 | 16 | >10% drop in delta EELI ROI 3-4 (dorsal) |
| 3 | 20 | 6 | 14 | >10% drop in delta EELI ROI 3-4 (dorsal) |
| 4 | 20 | 10 | 18 | >10% drop in delta EELI ROI 2-3-4 **and** SpO_2_ < 88%. |
| 5 | 20 | 8 | 14 | >10% drop in delta EELI ROI 3-4 (dorsal) |
| 6 | 20 | 8 | 14 | >10% drop in delta EELI ROI 4 (dorsal) |
| 7 | 18 | 5 | 14 | PEEP of 5 cmH2O was reached |
| 8 | 20 | 8 | 16 | SpO_2_ < 88%. |
| 9 | 20 | 8 | 16 | SpO_2_ < 88%. |
| 10 | 20 | 10 | 14 | SpO_2_ < 88%. |
| 11 | 20 | 8 | 14 | >10% drop in delta EELI ROI 4 (dorsal) |
| 12 | 20 | 5 | 8 | PEEP of 5 cmH2O was reached |
